# Functional impairment related to painful physical symptoms in patients with generalized anxiety disorder with or without comorbid major depressive disorder: post hoc analysis of a cross-sectional study

**DOI:** 10.1186/1471-244X-11-69

**Published:** 2011-04-21

**Authors:** Irene Romera, Ángel L Montejo, Fernando Caballero, Luis Caballero, José Arbesú, Pepa Polavieja, Durisala Desaiah, Inmaculada Gilaberte

**Affiliations:** 1 Clinical Research Department, Lilly, SA, Avenida de la Industria, 30, 28108 Alcobendas, Spain; 2 Hospital Universitario de Salamanca. School of Medicine, University of Salamanca, 37007 Salamanca, Spain; 3 Primary Care Research Department, 6th Health Area, Servicio Madrileño de Salud, 28020 Madrid, Spain; 4 Psychiatry Department, Hospital Puerta de Hierro, 28222 Madrid, Spain; 5 Primary Care Department, Centro de Salud de la Ería, 33013 Oviedo, Spain; 6 Consultant Scientific Communications and Training Lead, MCH - 92, Drop Code - 6122 Eli Lilly and Company, Lilly Corporate Center, Indianapolis, IN 46285, USA

## Abstract

**Background:**

Generalized anxiety disorder (GAD) is the most frequent anxiety disorder in primary care patients. It is known that painful physical symptoms (PPS) are associated with GAD, regardless the presence of comorbid major depressive disorder (MDD). However the specific role of such symptoms in patients' functional impairment is not well understood. The objective of the present study is to assess functional impairment related to the presence of PPS in patients with GAD.

**Methods:**

This is a post hoc analysis of a cross-sectional study. Functioning, in the presence (overall pain score >30; Visual Analog Scale) or absence of PPS, was assessed using the Sheehan Disability Scale (SDS) in three groups of patients; 1) GAD and comorbid MDD (GAD+MDD+), 2) GAD without comorbid MDD (GAD+MDD-), 3) controls (GAD-MDD-). ANCOVA models were used.

**Results:**

Of those patients with GAD+MDD+ (n = 559), 436 (78.0%) had PPS, compared with GAD+MDD- (249 of 422, 59%) and controls (95 of 336, 28.3%). Functioning worsened in both GAD groups in presence of PPS (SDS least squares mean total score: 16.1 vs. 9.8, p < 0.0001, GAD+MDD+; 14.3 vs. 8.2, p < 0.0001, GAD+MDD-). The presence of PPS was significantly associated with less productivity.

**Conclusions:**

Functional impairment related to the presence of PPS was relevant. Clinical implications should be considered.

## Background

Generalized anxiety disorder (GAD), a mental disorder highly prevalent in primary care patients (8%-14%), is generally associated with a significant impairment in patients' functioning [1,2]. Patients with GAD experience functional impairments, such as diminished social relationships, poorer well-being, and less satisfaction with life [3], that lead to reduced quality of life in the areas of interaction with friends, self-realization, subjective well-being [4,5], and work [6]. These patients are more likely to have absences from work and short-term disabilities; therefore, the indirect costs of the disease attributable to low productivity are increased [7]. The level of impairment is substantial and is even comparable to that of major depression [8]. As expected, the highest level of functional impairment occurs when GAD is comorbid with depression, which is a common feature in primary care [1,8].

The pathological feature most uniquely associated with GAD and that differentiates it from other anxiety disorders is excessive, pervasive, and uncontrollable worrying [9-12]. Such worrying in patients with GAD is frequently accompanied by a host of psychiatric symptoms, including irritability, restlessness, and concentration difficulties. Patients also experience a range of somatic symptoms such as cold clammy hands, dry mouth, sweating, nausea, and diarrhea [13]. Additionally, patients with GAD often complain about muscle pain and aches [14]. One recent study found that painful physical symptoms (PPS) in primary care patients with GAD were twice as prevalent as in a control group and the presence of comorbid major depressive disorder (MDD) further increased their prevalence [8]. Another recent study [15] using a community sample (N = 4181) showed a stronger association between GAD and pain (odds ratio, OR = 5.8 pain symptoms; OR = 16.0 pain disorder) compared to other anxiety disorders (OR = 2.4 pain symptoms; OR = 4.0 pain disorder).

Understanding the relationship between PPS and functional impairment in GAD could help clinicians to effectively manage these patients' treatment. Taking into account the poor treatment outcomes for GAD in terms of remission [16], it is clear that improved management of the disease is even more important. However, only a limited number of studies have focused on the specific role of PPS in functional and quality of life impairment in "real life" primary care patients, out of a clinical trial setting. As reported in a recent review, much of the research to date has focused on the prevalence of anxiety disorders in samples of patients that report chronic pain [17]. A small number of studies have assessed pain in patients with anxiety disorders, but most of them were limited to panic disorder and posttraumatic stress disorder and focused on chronic pain rather than PPS [18].

Our hypothesis is that the presence of PPS is associated with relevant and substantial functional- and health-related quality of life impairment in primary care patients with GAD, regardless of the presence of comorbid MDD. Previous studies have shown a significant deleterious effect of PPS on quality of life and functioning in patients with depression [19-22]. Therefore, the present study evaluates functional impairment associated with PPS patients with GAD, with or without MDD, in a primary care setting.

## Methods

### Study design

**Figure 1:**
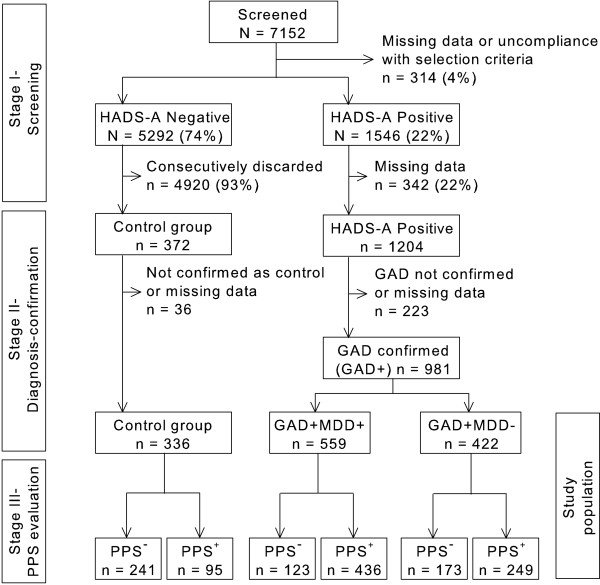
**Patient disposition**. Abbreviations: HADS-A = Hospital Anxiety and Depression Scale-Anxiety subscale; GAD = Generalized anxiety disorder; MDD = Major depressive disorder; PPS = Painful physical symptoms.

This is a post hoc analysis of a cross-sectional, multicenter epidemiological study that evaluated the prevalence of PPS in GAD patients with or without MDD [8]. Briefly, the study was carried out in a primary care setting covering 87 sites in Spain during April-June, 2007. The study had 3 stages in its design, including a consecutive screening to identify high-risk patients for GAD, a diagnosis confirmation of GAD along with an evaluation of the presence or absence of comorbid MDD, and a clinical evaluation for the presence of PPS (Figure 1).

### Eligibility criteria and study procedure

#### Consecutive screening stage

Patients ≥18 years of age who presented at primary care centres for any reason were consecutively screened for GAD using the Hospital Anxiety and Depression Scale-Anxiety subscale (HADS-A) [23]. Patients with a score of ≥8 were defined as positive and those with a score of <8 were defined as negative for GAD and consecutively selected as controls.

#### Diagnosis confirmation stage

Subsequent confirmation of the GAD diagnosis and evaluation for the presence or absence of comorbid MDD was administered per Diagnostic and Statistical Manual of Mental Disorders-Fourth Edition (DSM-IV) criteria by means of the Mini International Neuropsychiatric Interview (MINI) [13,24]. Controls, consecutively selected in the previous stage, were confirmed by a Hospital Anxiety and Depression Scale (HADS) total score of <11.

#### Clinical evaluation for the presence of PPS stage

After the first and second stages, three groups of patients were identified: 1) patients with GAD and comorbid MDD (GAD+MDD+); 2) patients with GAD without comorbid MDD (GAD+MDD-); and 3) controls (GAD-MDD-). Afterwards, the presence of PPS was clinically evaluated in these three groups of patients, who were classified as having or not having PPS (PPS^+ ^and PPS^-^, respectively). Ultimately, six groups of patients were categorized, combining diagnoses and presence of pain (Figure 1).

#### Exclusion criteria

Patients demonstrating any condition that would impede their understanding of the study were excluded.

#### Ethical and informed consent

Prior to the collection of data, all patients were informed about the purpose and objectives of the study and voluntarily provided written informed consent. The local ethical review board of the Hospital de Salamanca provided approval of the study protocol (F1J-XM-B024) as being in compliance with the Helsinki Declaration.

### Patient assessment

PPS were assessed by the Visual Analog Scale (VAS) for pain, which consists of six questions including overall pain, headache, back pain, shoulder pain, pain interference with daily activities, and time experiencing pain while awake [25]. The VAS overall pain severity score of >30 considered as the cutoff point in the identification of clinically significant PPS in patients with GAD [26], was adopted for identifying the presence of PPS (PPS^+^).

The Sheehan Disability Scale (SDS) was completed by the patient and used to assess functional status [27]. Using the SDS, 3 areas of the patient's life were scored: work, social life, and family life/home responsibilities. Response categories for each of these 3 areas had a score range from 0 to 10. A higher score corresponds to a greater degree of disruption and impairment in each area of life, with a maximum score of 30. In addition, the SDS includes questions about the number of lost days and the number of unproductive days during the prior week.

The EuroQoL-5D (EQ-5D), a standardized instrument [28] that measures health outcomes, was used to assess quality of life. It provides a simple descriptive profile and single index values for health status. The EQ-5D has 5 domains: mobility, self-care, usual activities, pain/discomfort, and anxiety/depression. Scoring is on a 3-point scale, with self-completed response records obtained from individual patients. Lower scores indicate poorer health status.

The Clinical Global Impression of Severity (CGI-S) scale [29] was used to assess the severity of the patient's symptoms. The CGI-S scale is a single-item rating of the clinician's assessment of the patient's symptoms severity. The severity score ranges from 1 (normal, not at all ill) to 7 (among the most extremely ill patients).

Other assessments (age, gender, education level, work status, current medical and psychiatric co-morbidities and existing treatments) were based on direct questioning to the patient and patient's clinical history. Checklists with the common co-morbidities were provided to the investigators.

### Statistical analysis

Sample size was estimated for the primary objective of the study, which was to assess the prevalence of PPS in GAD patients (GAD+MDD-) as compared to GAD patients with comorbid MDD (GAD+MDD+) and controls (GAD-MDD-) [8]. Therefore, it was estimated in order to achieve a power of 88% when comparing differences between a proportion of patients with PPS in the GAD+MDD- group of 44% [30] and a proportion of patients with PPS in the control group of 30% [31]. On the other hand, the study would have a 80% power to detect the difference between a proportion of patients with PPS of 44% in the GAD+MDD- group and a proportion of patients with PPS of 57% in the GAD+MDD+ group [32] with the following sample sizes: 374 in the GAD+MDD- group, 249 in the GAD+MDD+ group, and 249 in the control group.

In previous studies [1], the prevalence of comorbid MDD in patients with GAD was approximately 40% and the prevalence of GAD in primary care patients was around 8%. In order to obtain a final sample size of 249 patients in the comorbid group (GAD+MDD+), screening of 8650 patients was performed in order to adjust for a 10% rate of refusal to participate among patients who were invited to be screened.

Comparisons between patient groups regarding demographics, clinical characteristics, and current medications were made by means of a chi-square test for qualitative variables and by analysis of variance (ANOVA) for quantitative variables.

The associations between the presence of PPS and functioning in the different groups of diagnoses (GAD+MDD+; GAD+MDD-; control) were analyzed using an analysis of covariance (ANCOVA) model with the independent fixed effects combination of diagnosis and pain (categorized at six levels: the three different diagnoses combined with having pain or not), gender, age, medical, psychiatric comorbidities, and the CGI-S, interactions were not included. The dependent variable was the SDS total score. If the main effects were significant, pair wise comparisons between all categories in that particular effect were performed.

In order to evaluate the association between the presence of PPS and the number of underproductive days in the three different groups of diagnoses, a second ANCOVA model was developed including the same terms as well as the interaction age and diagnosis. The dependent variable was the number of underproductive days in the prior week, as measured by the SDS.

Finally, to evaluate the association between the presence of PPS and health status in the three different groups of diagnoses, a third ANCOVA model was developed including the same terms, with the dependent variable being the EQ-5D score. UK population norms were used for standardizing the EQ-5D score.

All statistical analyses were performed using SAS software (version 8.2).

## Results

### Patient disposition

Of the total 7152 screened patients, 1546 (22%) tested positive for GAD and subsequently 981 (13.7%) were confirmed as having a diagnosis of GAD. Of those patients, 559 (57%) had GAD with comorbid MDD (GAD+MDD+) and a large percentage of them had PPS (78%, n = 436). Of the 422 (43%) patients with GAD but without comorbid MDD (GAD+MDD-), 249 (59%) had PPS. Of the remaining 5292 patients who screened negative, 336 were selected and confirmed as controls and 95 (28%) of them had PPS (Figure 1).

### Patients demographics and clinical characteristics

The mean (standard deviation: SD) age (years) was 52.2 (14.7) for GAD+MDD+ patients, 49.8 (14.5) for GAD+MDD- patients, and 50.8 (17.3) for control patients (Table 1). Patients with PPS were slightly older than those without PPS (ANOVA p-value ≤0.05, PPS^+ ^vs. PPS^-^, for all groups: GAD+MDD+, GAD+MDD-, and control patients). Medical and psychiatric comorbidities were more prevalent in the presence of PPS in all the three different groups of diagnoses, but particularly in patients with GAD and comorbid MDD.

**Table 1:** Demographics and clinical characteristics

	GAD+MDD+ (N = 559)		GAD+MDD- (N = 422)		Control (N = 336)	
			
	PPS^+^ (N = 436)	PPS^-^ (N = 123)	PPS^+^ (N = 249)	PPS^-^ (N = 173)	PPS^+^ (N = 95)	PPS^-^ (N = 241)
Gender, female, n (%)*	338 (77.5)	95 (77.2)	196 (78.7)^b^	118 (68.2)	64 (67.4)^c^	119 (49.4)
Age (years), mean (SD)**	53.3 (14.7)^d^	48.3 (13.8)	51.5 (14.7)^d^	47.4 (13.8)	54.4 (17.4)^d^	49.4 (17.1)
**Medical comorbidities, n (%)**						
At least one*	329 (75.5)^a^	59 (48.0)	187 (75.1)^b^	81 (46.8)	66 (69.5)^c^	122 (50.6)
**Hypertension^†^**	156 (35.8)^a^	29 (23.6)	76 (30.5)	38 (22.0)	28 (29.5)	72 (29.9)
**Diabetes mellitus^††^**	56 (12.8)^a^	6 (4.9)	28 (11.2)	12 (6.9)	7 (7.4)	18 (7.5)
**Coronary artery disease^‡^**	29 (6.7)	4 (3.3)	8 (3.2)	3 (1.7)	4 (4.2)	8 (3.3)
Degenerative osteoarthritis*	116 (26.6)^a^	10 (8.1)	61 (24.5)^b^	21 (12.1)	29 (30.5)^c^	25 (10.4)
**Psychiatric comorbidities, n (%)**						
At least one*	146 (33.5)^a^	28 (22.8)	62 (24.9)	30 (17.3)	4 (4.2)	3 (1.2)
CGI-Severity, mean (SD)**	4.5 (0.9)^d^	3.8 (1.0)	3.6 (1.0)^d^	2.9 (1.1)	2.0 (1.2)^d^	1.3 (0.7)
**Current medication, n (%)**						
Antidepressants*	153 (35.1)	43 (35.0)	44 (17.7)	30 (17.3)	2 (2.1)	4 (1.7)
Benzodiazepines*	205 (47.0)^a^	42 (34.1)	102 (41.0)^b^	46 (26.6)	8 (8.4)	10 (4.1)
Analgesics*	265 (60.8)^a^	36 (29.3)	140 (56.2)^b^	36 (20.8)	55 (57.9)^c^	38 (15.8)
NSAIDs*	168 (38.5)^a^	21 (17.1)	96 (38.6)^b^	32 (18.5)	37 (38.9)^c^	18 (7.5)
Analgesic/NSAIDs*	301 (69.0)^a^	44 (35.8)	162 (65.1)^b^	53(30.6)	66 (69.5)^c^	46 (19.1)

GAD = Generalized anxiety disorder; MDD = Major depressive disorder; PPS = Painful physical symptoms; SD = Standard deviation; CGI = Clinical Global Impression; NSAIDs = Non-steroidal anti-inflammatory drugs; ANOVA = Analysis of variance; *Chi-square test p ≤ 0.0001; **ANOVA p ≤ 0.0001; †Chi-square test p = 0.0123; ††Chi-square test p = 0.0298; ‡Chi-square test p = 0.0662; ^a^Chi-square p ≤ 0.05 vs. GAD+MDD+ PPS**^- ^**patients; ^b^Chi-square p ≤ 0.05 vs. GAD+MDD-PPS**^- ^**patients; ^c^Chi-square p ≤ 0.05 vs. controls PPS**^- ^**patients; ^d^ANOVA p ≤ 0.05 vs. PPS**^- ^**patients.

### Concomitant medications

Regarding the use of psychotropic medication, 35.1% of GAD+MDD+ patients and 17.5% of GAD+MDD- patients were using some type of antidepressant drug, with no significant differences between PPS^+ ^and PPS^- ^patients. However, a more frequent use of benzodiazepines was reported particularly in PPS^+ ^patients (for GAD+MDD+ patients: 47.0% vs. 34.1%, p = 0.0111, PPS^+ ^vs. PPS^- ^respectively; for GAD+MDD- patients: 41.0% vs. 26.6%, p = 0.0023, PPS^+ ^vs. PPS^-^, respectively). As expected, a significantly higher number of patients with GAD and PPS were taking analgesic or anti-inflammatory agents (69.0% and 65.1% for GAD+MDD+ and GAD+MDD-, respectively), compared to those without PPS (35.8% and 30.6%, respectively; p < 0.0001 for both comparisons).

### Functional impairment related to the presence of PPS

**Figure 2:**
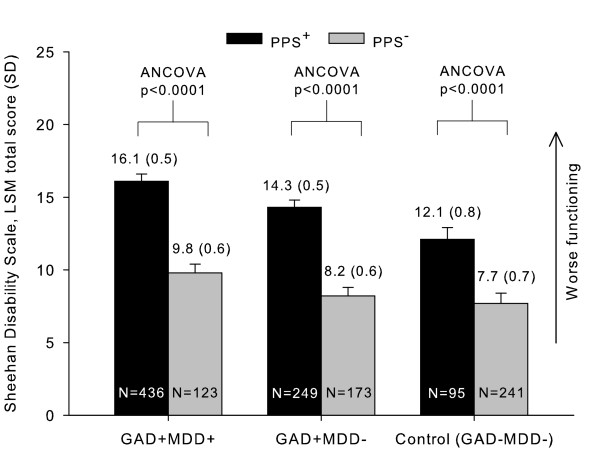
**Functioning (SDS total score) by the presence of PPS in patients with GAD, with or without co-morbid MDD**. Abbreviations: GAD = Generalized anxiety disorder; MDD = Major depressive disorder; PPS = Painful physical symptoms; SDS = Sheehan Disability Scale, SD = Standard deviation; LSM = Least square mean.

Patients with PPS in all groups exhibited significantly worse functional impairment compared to patients without PPS, as expressed by a significantly higher SDS total score (F-value = 52.8 for the combination of diagnoses and pain; p < 0.0001, PPS^+ ^vs. PPS^- ^for GAD+MDD+, GAD+MDD-, and control groups). This result indicates that the presence of PPS contributes to worse functional impairment in work, social, and family life, when adjusted by patients' severity (CGI-S scale), age, gender, or comorbidity, (Figure 2). As expected, functional impairment was worst in patients suffering from GAD and comorbid MDD and PPS (SDS total score least squares means [LSM] [SD] = 16.1 [0.5] p < 0.0001 vs. GAD+MDD- and vs. control groups, regardless of the presence of PPS). Interestingly, the magnitude of the functioning impairment related to the presence of PPS was relevant as it was >1.5 fold worse when PPS was present (SDS total score LSM [SD] in GAD+MDD+ patients = 16.1 [0.5] vs. 9.8 [0.6]; p < 0.0001, PPS^+ ^vs. PPS^-^; SDS total score LSM [SD] in GAD+MDD- patients = 14.3 [0.5] vs. 8.2 [0.6]; p < 0.0001, PPS^+ ^vs. PPS^- ^; SDS total score LSM [SD] in controls = 12.1 [0.8] vs. 7.7 [0.7]; p < 0.0001, PPS^+ ^vs. PPS^-^). Other factors that significantly contributed to patients' functional impairment were: age (F-value = 21.9; p < 0.0001), being female (F-value = 5.0; p = 0.0253), medical comorbidity (F-value = 5.6; p = 0.0180), and patients' severity as measured by the CGI-S scale (F-value = 33.6; p < 0.0001).

### Underproductive days related to the presence of PPS

**Figure 3:**
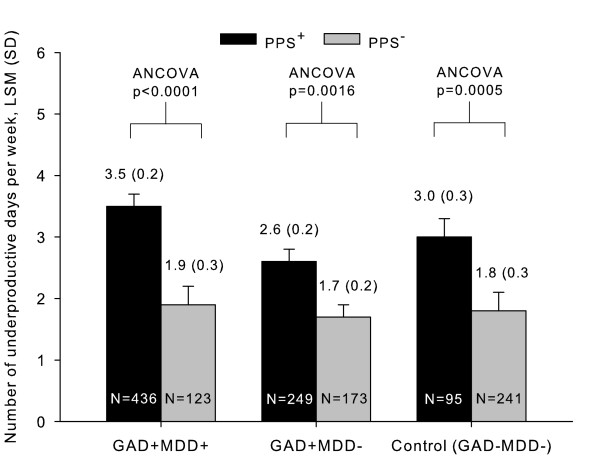
**Underproductive days/weeks by the presence of PPS in patients with GAD, with or without comorbid MDD**. Abbreviations: GAD = Generalized anxiety disorder; MDD = Major depressive disorder; PPS = Painful physical symptoms, SD = Standard deviation; LSM = Least square mean; ANCOVA = Analysis of covariance.

Analysis of underproductive days per week showed that patients with PPS in all the groups experienced a significantly higher number of underproductive days compared to patients without PPS (F-value = 6.1 for the combination of diagnoses and pain p < 0.0001, p = 0.0016, p = 0.0005; PPS^+ ^vs. PPS^- ^for GAD+MDD+, GAD+MDD-, and control groups, respectively) (Figure 3). The presence of PPS in GAD+MDD+ patients was associated with a nearly two-fold increase in the number of underproductive days (LSM [SD] = 3.5 [0.2] vs. 1.9 [0.3]; p < 0.0001, PPS^+ ^vs. PPS^-^). Interestingly, in the absence of PPS, no statistically significant differences between study groups (GAD+MDD+, GAD+MDD-, control) were found regarding the number of underproductive days. Finally, other factors found to be related to more underproductive days were patients' severity measured by CGI-S scale (F-value = 12.4; p < 0.0001) and the interaction between age and diagnosis (F-value = 3.4; p = 0.0049). This means that the effect of diagnosis on the number of unproductive days varies according to age. Gender, medical comorbidity, and psychiatric comorbidity were not found to be significantly associated with underproductive days.

### Quality of life related to the presence of PPS

**Figure 4:**
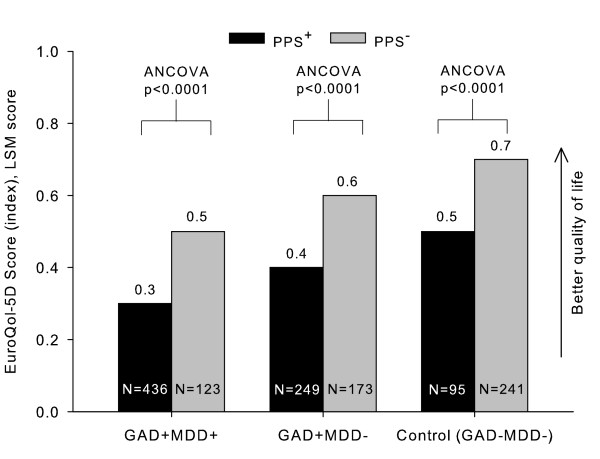
**Quality of life (EuroQoL-5D total score) by the presence of PPS in patients with GAD, with or without comorbid MDD**. Abbreviations: GAD = Generalized anxiety disorder; MDD = Major depressive disorder; PPS = Painful physical symptoms; SD = Standard deviation.

The quality of life as assessed by the EQ-5D score showed that patients with PPS in all studied groups (viz. GAD with comorbid MDD, GAD without MDD, and control groups) experienced a significantly decreased quality of life as compared to patients without PPS (F-value = 30.3 for the combination of diagnoses and pain, p < 0.0001; PPS^+ ^vs. PPS^- ^for GAD+MDD+, GAD+MDD-, and control groups) (Figure 4). Similarly to functionality, the poorest quality of life was found in PPS positive GAD patients with comorbid MDD (LSM [SD] = 0.3 [0.0], p < 0.0001 vs. GAD+MDD- and control groups, with or without PPS). Results suggest that the presence of PPS alone contributes to additional decrease in quality of life. Other factors found to be significantly related to a lower quality of life were age (F-value = 8.1; p = 0.0044), medical comorbidity (F-value = 6.0; p = 0.0147), and patients' severity assessed by the CGI-S scale (F-value = 44.3; p < 0.0001). Gender and psychiatric comorbidity were not significantly related (p = 0.1925 and p = 0.6363, respectively).

## Discussion

This epidemiological study shows that PPS are a common feature in patients suffering from GAD, especially in those presenting comorbid MDD [8]. In this post hoc analysis, the presence of PPS was clinically and statistically significantly related to worse patients' functioning, productivity, and quality of life regardless of the patient's disease severity, age, gender, or comorbidity. The magnitude of this association between the presence of PPS and functioning impairment has proven to be relevant and persists even in the absence of comorbid MDD. Thus, the presence of PPS in patients with GAD, with or without comorbid MDD, was shown to be strongly associated with functional impairment in patient's work, social, and family lives. Hence, it is highly relevant for the physician to assess patients for PPS when evaluating for and treating GAD, regardless of the presence of comorbid MDD. A comprehensive evaluation of the role of these symptoms in a patient's ability to perform daily activities could improve the management of GAD and ultimately the patient's outcome.

The present study is the first to report the results of an assessment of functional impairment related to the presence of PPS in a large sample of primary care patients with GAD. The few similar published studies to date were either focused on community samples [33] or included patients suffering from any anxiety disorder other than GAD [34]. Our results are consistent with these previous studies, in which pain symptoms were reported to be associated with poorer functioning in patients suffering from social anxiety and post-traumatic stress disorder [35] as well as in a community sample diagnosed with any anxiety disorder [33,34]. However, contrary to the results of Means-Christensen et al. [35], our study shows a clear association between PPS and a diagnosis of anxiety disorder (GAD in our study) that is not mediated by the presence of comorbid MDD. This could be due to the differences in patient samples and designs of both studies.

It is known that GAD is associated with clinically significant impairments in social, occupational, and/or family functioning [1]. As reported previously [1,2,36,37], several factors that contribute to greater functional impairment were determined, including the presence of psychiatric or medical comorbidity, age, patients' disease severity, and being female. However, none of the available studies on GAD focused on the effects of PPS on functional impairment. The current study contributes to the understanding of the specific role of such pain symptoms in worsening the functioning in patients with GAD. The presence of PPS is associated with a statistically and clinically significant impairment in patients' functioning, productivity, and quality of life, regardless of patients' disease severity, age, gender, or MDD comorbidity. In regard to productivity, patients with PPS had substantially more underproductive days per week compared to those without PPS. As expected, the presence of comorbid MDD was associated with worse productivity [38].

Several clinical implications could arise from these study results; PPS are frequent and significantly interfere with patients' family, social, and work activities. In order to ensure effective management of patients with GAD, it would be desirable to pay special attention not only to the typical symptoms of the disease but also to these PPS. Therefore, clinicians may routinely evaluate the extent to which symptoms impact patients' ability to perform well in a range of activities or areas. However, this task may not be as easy as it appears, given that functioning and pain symptoms are not commonly or systematically measured during the management of the disease. Also, primary care physicians do not always make the association between pain symptoms and mental disorders; that is, they tend to associate pain symptoms more with a somatic disease rather than a mental disorder [31]. Primary care physicians may have to bear in mind that PPS are very commonly associated with both depression [39-43] and anxiety [8,32-34]. Moreover, they should be aware that that their presence could be associated with poorer treatment outcome and be a barrier to an adequate diagnosis of the disease [42,44,45].

In accordance with current knowledge, this study found that GAD was not recognized by the physician for a large proportion of primary care patients, thus a large percentage of patients did not receive appropriate treatment for the disease [46]. Among the treatment options, only one-third of patients (or even fewer) was receiving antidepressant treatment, while benzodiazepines were being used predominantly. Awareness or recognition and adequate treatment of GAD would be important, particularly in primary care settings. The fact that patients with emotional distress report physical or somatic symptoms more often than psychological symptoms [47] could be a contributing factor for the under-recognition rates found in this study [8]. In order to improve the recognition of GAD in primary care, physicians should see these somatic symptoms as a high-risk factor not only for depression, but also for anxiety, especially if the symptoms are multiple and medically unexplained [42].

In the light of the study findings, primary care physicians should seek for an effective management of these PPS, and closely monitor the patients' improvement, so in this way the chances of functional recovery would be increased.

The present analysis has several limitations; the main limitation is that the analysis is focused mainly on correlations and associations, but does not provide any causal relationships. The results of the analysis cannot rule out possibility of patients' recall bias associated with the condition of underproductive days. However, the presence of a control group mitigates that bias. It could be argued that the use of categorical research-based criteria, such as the DSM-IV, is not useful in clinical practice because it artificially separates depression from anxiety. However, their use in this study is recommended in order to fully understand the specific role of pain symptoms in anxiety that is not mediated by the presence of MDD. Moreover, using standard criteria may allow further comparison with other research. The results should be not generalized to patients treated by psychiatrists, inpatients or other populations. Controls were selected based on the HADS-A subscale and total score on the HADS, and no structured interview was carried out. However, negative predictive values for selected cutoff points are 92% (HADS-A) and 78% (HADS) [48]. The major strength of this analysis is that it is the first time the specific role of PPS in functional impairment and their influence on underproductivity and quality of life was analyzed in a large sample of primary care patients with GAD, with or without MDD, by means of a controlled-design study. The naturalistic study design and the representativeness of the sample allow the generalization of the results to primary care patients with GAD. The presence of a control group of patients without MDD or GAD allows a more conclusive and comprehensive interpretation of the results.

## Conclusions

The results of this study demonstrate a significant association of functional impairment with the presence of PPS in GAD patients, with or without comorbid MDD. Furthermore, the patients with PPS showed poorer quality of life. The frequent occurrence of PPS and their impact on patients' daily activities suggests that there is a need for accurate clinical diagnosis and management of PPS while evaluating and treating patients with GAD.

## Competing interests

Drs. Romera, Gilaberte, Ms Polavieja are full time employees of Lilly SA. Dr. Desaiah was a full time employee of Eli Lilly and Company, Indianapolis, IN, USA during the conduct of the study. Dr. Arbesú has received grant support from Lilly SA; and has served as a consultant for and/or on advisory boards for Lilly SA; and has served as a speaker for Lilly SA, Lundbeck, Wyeth, Esteve, Almirall, Pfizer, Mylan and GSK. Dr. Montejo has received grant support from Eli Lilly, BMS-Otsuka, Lundbeck, Pfizer, AstraZeneca, Sanofi and GSK; has received honoraria from Servier, Eli Lilly, BMS-Otsuka. GSK, Sanofi, AstraZeneca, Boehriger and Wyeth; has served as a consultant for and/or on advisory boards for Eli Lilly, BI, GlaxoSmithKline, Servier and AstraZeneca.

## Authors' contributions

IR, ALM, FC, LC, JA, PP and IG, have been involved in the study design, interpretation of the data, revision of the manuscript and the decision to submit the manuscript for publication. IR and DD have been involved in interpretation of the data and writing the manuscript. All authors have been involved in the decision to submit the manuscript for publication and have read and approved the final manuscript.

## Pre-publication history

The pre-publication history for this paper can be accessed here:

http://www.biomedcentral.com/1471-244X/11/69/prepub
